# Development and physiological effects of an artificial diet for *Wolbachia*-infected *Aedes aegypti*

**DOI:** 10.1038/s41598-017-16045-6

**Published:** 2017-11-16

**Authors:** Heverton Leandro Carneiro Dutra, Silvia Lomeu Rodrigues, Simone Brutman Mansur, Sofia Pimenta de Oliveira, Eric Pearce Caragata, Luciano Andrade Moreira

**Affiliations:** 1 Endossimbiontes e Interação Patógeno-Vetor, Centro de Pesquisas René Rachou - Fiocruz, Belo Horizonte, MG 30190-002 Brazil; 2 Present Address: Department of Immunology and Infectious Diseases, Harvard T. H. Chan School of Public Health, Boston, Massachusetts United States of America; 3 Present Address: W. Harry Feinstone Department of Molecular Microbiology and Immunology, Johns Hopkins Bloomberg School of Public Health, Baltimore, Maryland United States of America

## Abstract

The endosymbiotic bacterium *Wolbachia* spreads rapidly through populations of *Aedes aegypti* mosquitoes, and strongly inhibits infection with key human pathogens including the dengue and Zika viruses. Mosquito control programs aimed at limiting transmission of these viruses are ongoing in multiple countries, yet there is a dearth of mass rearing infrastructure specific to *Wolbachia*-infected mosquitoes. One example is the lack of a blood meal substitute, which accounts for the *Wolbachia*-specific physiological changes in infected mosquitoes, that allows the bacterium to spread, and block viral infections. To that end, we have developed a blood meal substitute specifically for mosquitoes infected with the *w*Mel *Wolbachia* strain. This diet, ADM, contains milk protein, and infant formula, dissolved in a mixture of bovine red blood cells and Aedes physiological saline, with ATP as a phagostimulant. Feeding with ADM leads to high levels of viable egg production, but also does not affect key *Wolbachia* parameters including, bacterial density, cytoplasmic incompatibility, or resistance to infection with Zika virus. ADM represents an effective substitute for human blood, which could potentially be used for the mass rearing of *w*Mel-infected *A. aegypti*, and could easily be optimized in the future to improve performance.

## Introduction

*Aedes aegypti* mosquitoes infected with the *w*Mel strain of the bacterium *Wolbachia pipientis* are currently being deployed in several countries around the world as part of an extensive program to limit the transmission of mosquito-transmitted arboviruses, which have a serious impact on human health (http://tinyurl.com/Who-report-2016). *Wolbachia* infections in mosquitoes naturally possess several properties that could potentially make them effective agents of disease control. Firstly, they induce the reproductive manipulation cytoplasmic incompatibility (CI) - a type of reproductive incompatibility that limits viable egg production in *Wolbachia*-uninfected female mosquitoes, and thus promotes the spread of the bacterium in the field^1^. Secondly, the bacterium is maternally transmitted at very high rates^2,3^. Thirdly, the *w*Mel strain has minimal impact on host fitness^3,4^, which likely serves to make *w*Mel-infected *A. aegypti* highly competitive with wildtype mosquitoes in the field^1^. Finally, *w*Mel infection has a natural anti-pathogenic effect in mosquitoes, called pathogen interference or pathogen blocking, wherein *Wolbachia* limits the infection and replication of key viruses including dengue (DENV), chikungunya (CHIKV), and Zika (ZIKV) in mosquito tissues and saliva^3,5–8^.

The aim of the World Mosquito Program releases (www.worldmosquitoprogram.org) is to introduce *Wolbachia*-infected mosquitoes, which are less susceptible to arboviral infections, into areas with high levels of disease transmission. This transmission blocking approach to mosquito control does not require laborious sexing of pupae, as both males and females facilitate the spread of the bacterium. The bacterium is also naturally abundant amongst insect taxa^9,10^, and there is an established regulatory framework for the use of *Wolbachia* as a biological control agent of mosquitoes in multiple countries^11,12^.

Yet, as with any mosquito control program the implementation of *Wolbachia*-infected mosquitoes on a large scale involves potential logistical issues. One of the more important of these is the need to blood feed large numbers of mosquitoes in order to generate sufficient mosquito material for the releases. In some countries, obtaining large volumes of blood could be subject to both ethical and regulatory risk, while there is also the potential that human blood stocks could be contaminated with pathogens, including arboviruses that may circulate endemically in those regions, and that could lead to the accidental infection of colony mosquitoes.

For that reason, blood meal substitutes (BMS) have long been considered as an alternative to blood feeding to facilitate the mass rearing of mosquitoes^13,14^. The composition of these BMS can vary from a simple protein solution to complex mixtures of compounds, including blood-derived components^15,16^. These diets can be considered successful as they lead mosquitoes to produce large numbers of viable eggs after feeding. However they are often also expensive to purchase, or can contain components that are laborious to prepare, which can limit their usefulness, particularly in more remote settings where sophisticated laboratory equipment may not be available.

An additional complication arises when mosquitoes are infected with *Wolbachia*, as the symbiont is metabolically dependent on the host^17^ and on nutrients imbibed by the host. Infection with *w*Mel alters the expression of a wide range of genes involved in protein, sugar and lipid metabolism^18^, which suggests that infection is associated with perturbation of normal metabolic processes. *Wolbachia*-host metabolic interactions can affect immune response, fecundity, stress response, and metabolite levels^19–22^. Some *Wolbachia* strains also decrease host reproductive capacity, particularly when human blood is substituted for animal blood, which suggests that blood composition is critical to the fitness of *Wolbachia*-infected mosquitoes^23^. Consequently, it is possible that a BMS used in the context of releases may need to be specifically tailored to *Wolbachia*-infected mosquitoes in order to limit potential fitness effects.

A further complication is that *Wolbachia*-infected mosquitoes that are fed on a BMS would need to display comparable levels of CI and pathogen interference to those that are fed on blood, as these traits are critical for effective disease control in the field, post-release. Traits like pathogen interference are strongly linked to bacterial density^3^, which can change depending on the protein or carbohydrate content of the host diet^24,25^, and thus could foreseeably be affected by feeding on a BMS.

To that end, we have developed a BMS specific to *w*Mel-infected *A. aegypti* (ADM - Artificial diet for *w*Mel-infected *Aedes aegypti*), which contains low-cost, widely available materials. ADM is easy and quick to prepare, and promotes high fecundity and fertility amongst fed mosquitoes. We have also compared levels of *Wolbachia* maternal transmission, bacterial density, longevity, CI, and inhibition of ZIKV infection for *w*Mel-infected *A. aegypti* fed either human blood or ADM. We determined that ADM feeding does not significantly affect any of these key parameters, and is thus potentially suitable for use in mass rearing and mosquito control programs involving *Wolbachia*-infected mosquitoes.

## Results

### Blood fractions

**Figure 1:**
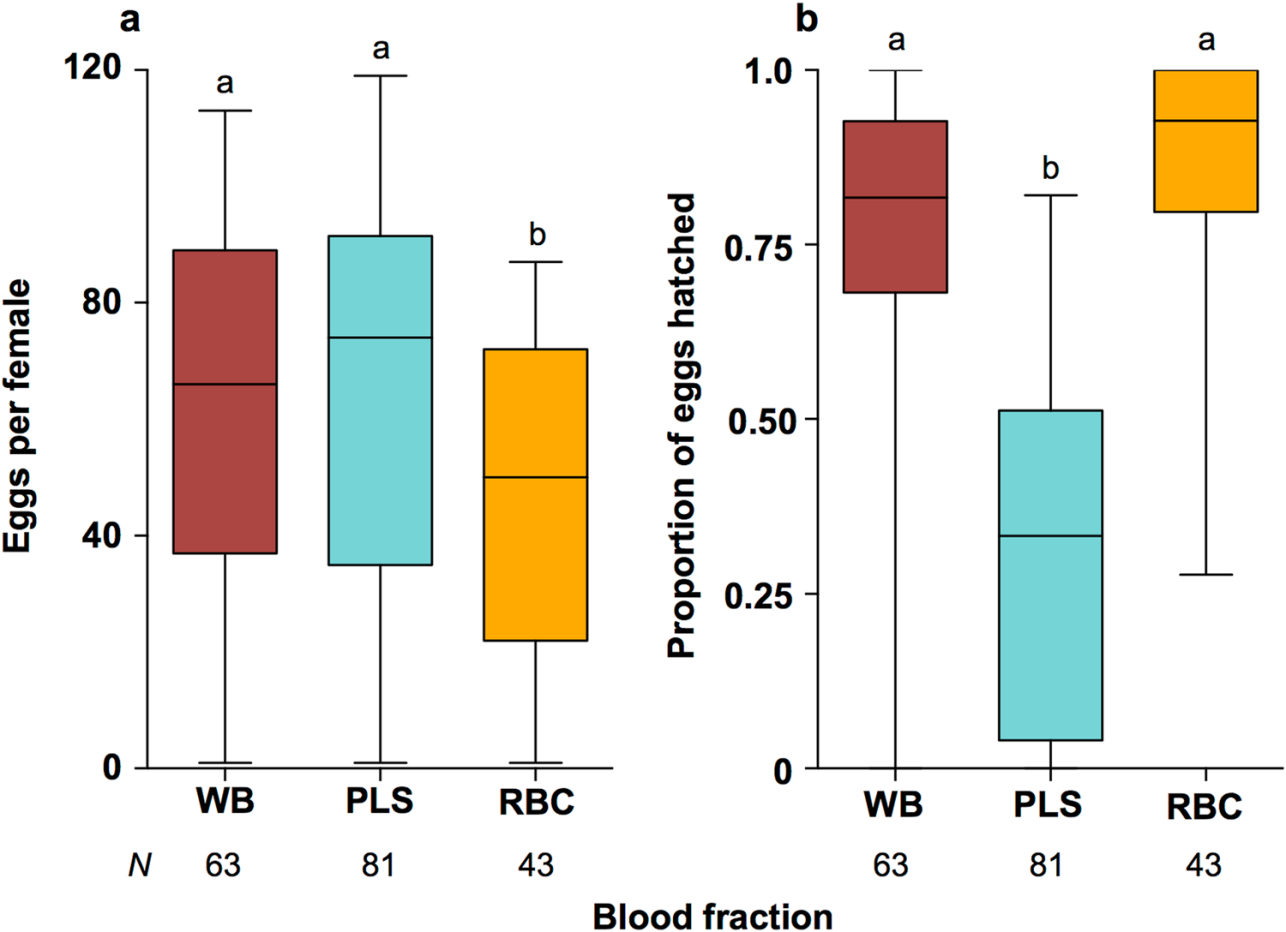
Fecundity and hatch rate for *w*Mel-infected *A. aegypti* fed on different blood fractions. Whole human blood (WB) was separated into plasma (PLS) and red blood cells (RBC) by centrifugation, and then fed to Mel mosquitoes. Fecundity (**a**) and hatch rate (**b**) were recorded for individual mosquitoes across 2 experiments. Fecundity levels were decreased after feeding only on RBC, while hatch rate was decreased after feeding only on PLS. Data were compared using Kruskal-Wallis ANOVA, and Dunn’s multiple comparisons test. Different letter codes represent statistically significant differences between treatments. Box - median and interquartile range. Whiskers - minimum and maximum.

We fed *w*Mel-infected *Ae. aegypti* with whole human blood (WB), plasma (PLS), or red blood cells (RBC) in order to determine which blood fractions were required for mosquitoes to produce large numbers of viable eggs. Median fecundity (number of eggs laid) was significantly higher for mosquitoes fed on either WB (66 eggs) or PLS (74) than for RBC (50) (Fig. 1a, Kruskal-Wallis: *H* = 11.35, *N* = 187, *P* < 0.01). In contrast, median hatch rates (percentage of eggs hatched) were significantly higher for WB (81.73%) and RBC (90.12%) than for PLS (28.21%) (Fig. 1b, Kruskal-Wallis: *H* = 89.51, *N* = 187, *P* < 0.0001), indicating that *w*Mel-infected mosquitoes required components from the PLS fraction to promote high fecundity, and components from the RBC fraction to promote high levels of fertility.

### Initial development

Based on a review of the literature^26^ and a small pilot experiment (See Supplementary File 1 and Supplementary Fig. 1), we selected Aedes physiological saline (APS) as the most promising solution to use as a solvent for our diet. We then investigated a number of inexpensive, unflavoured, (USD $20–25 per Kg), widely available sources of plant- (pea, rice and soy) and animal-derived (albumin from eggs, concentrated milk whey, and isolated milk whey) proteins typically used as nutritional supplements for exercise and bodybuilding. We then offered these proteins to Mel mosquitoes at either 150 or 200 mg/mL in order to compare rate of feeding, and fecundity and egg hatch levels to see if any were promising candidates for inclusion in an artificial diet (Table 1). No mosquitoes fed on the soy or albumin proteins, with these proteins displaying poor solubility in APS. A small number of mosquitoes fed on the pea protein, but 80% of these died prior to laying eggs and the remainder laid no eggs. Higher feeding rates were obtained for the rice and both milk proteins. However, only the concentrated milk protein (MC) produced reasonable numbers of eggs and viable larvae.

**Table 1:** Feeding, fecundity and fertility values for different protein types.

Protein	WB	Rice	Milk C	Milk I	Pea	Soy	Albumin
Feeding rate (%)	94	80	67	31	13	0	0
Fecundity	54	0	10	1	0	0	0
Fertility	37	0	4	0.5	0	0	0
Hatch rate (%)	77.94	0	23.61	9.80	0	0	0

WB = Human blood, Fecundity = Median eggs laid per female, Fertility = Median live larvae per female, Hatch rate = median % of eggs hatched. All diets except WB included ATP C_Final_ = 1 mM.

**Figure 2:**
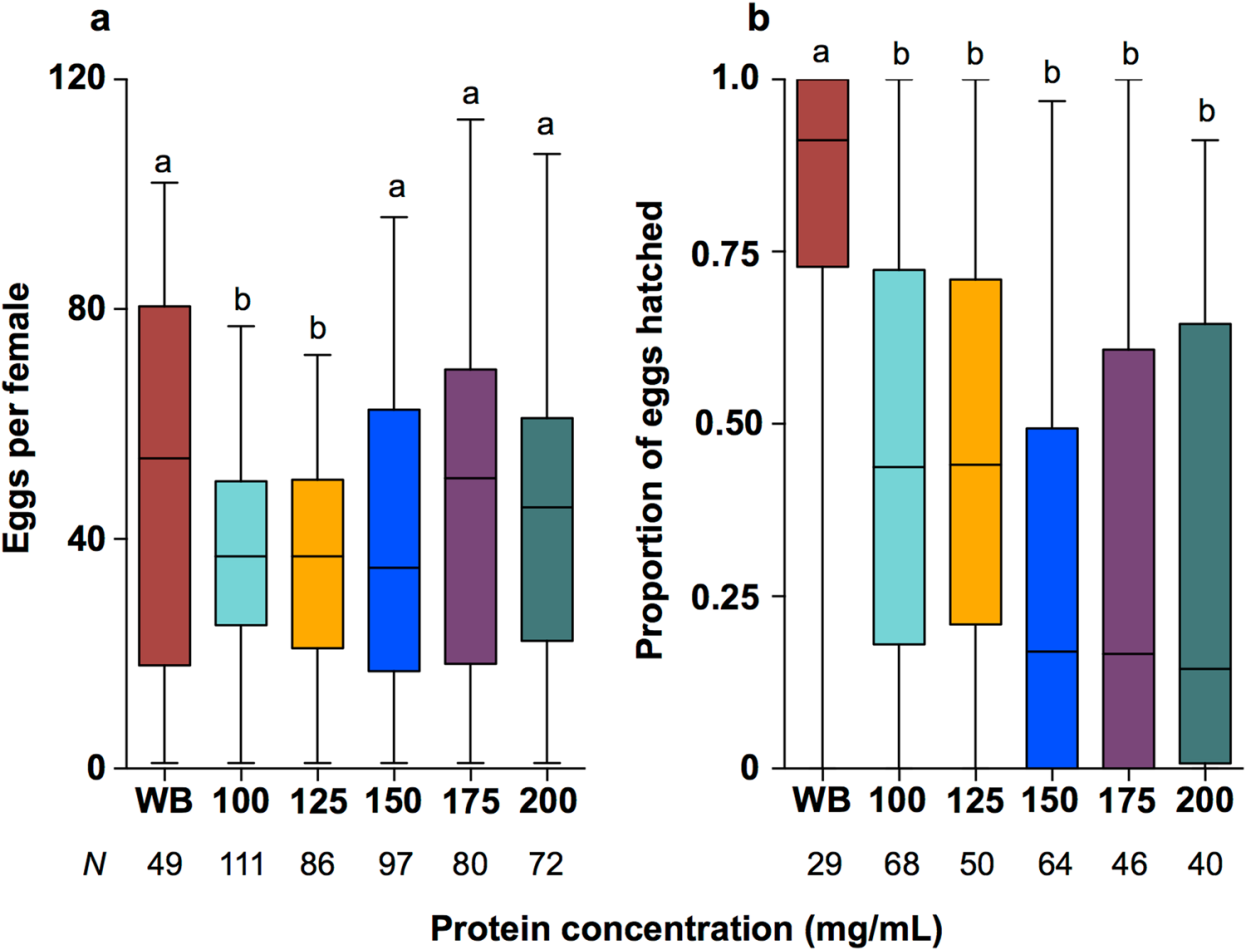
Fecundity and hatch rate for *w*Mel-infected *A. aegypti* fed on different concentrations of MC protein. Mel mosquitoes were fed either whole human blood (WB), or one of 5 concentrations of MC protein (100–200 mg/mL). Fecundity (**a**) and hatch rate (**b**) were recorded for individual mosquitoes. Higher MC protein concentrations (150, 175 & 200 mg/mL) produced greater levels of fecundity, but had greatly reduced hatch rates compared to WB or lower MC concentrations (100–125 mg/mL). Data were compared using Kruskal-Wallis ANOVA, and Dunn’s multiple comparisons test. Different letter codes represent statistically significant differences between treatments. Box - median and interquartile range. Whiskers - minimum and maximum.

We then performed range-finding assays, feeding MC at different concentrations (100–200 mg/mL) in order to determine the optimal feeding concentration. The fecundity resulting from MC concentrations of 150–200 mg/mL was not significantly different to fecundity of mosquitoes fed WB, and this was significantly higher than the fecundity of mosquitoes fed 100 or 125 mg/mL of MC (Fig. 2a, Kruskal-Wallis: *H* = 17.50, *N* = 495, *P* < 0.01). However, all MC concentrations produced a lower hatch rate than WB (Fig. 2b, Kruskal-Wallis; *H* = 59.27, *N* = 297, *P* < 0.0001). Comparison of median hatch rates amongst the MC diets revealed that the less concentrated diets performed better - 100 (43.74%), 125 (44.05%), 150 (16.99%), 175 (16.64%), and 200 (14.51%). This equated to between 7–8 additional larvae for the 125 diet based on treatment medians. Consequently, we selected the 125 mg/mL concentration for further testing. In further experiments we combined this concentration of protein with the blood fractions described above (See Supplementary File 1 and Supplementary Fig. 2).

### Additional components

**Figure 3:**
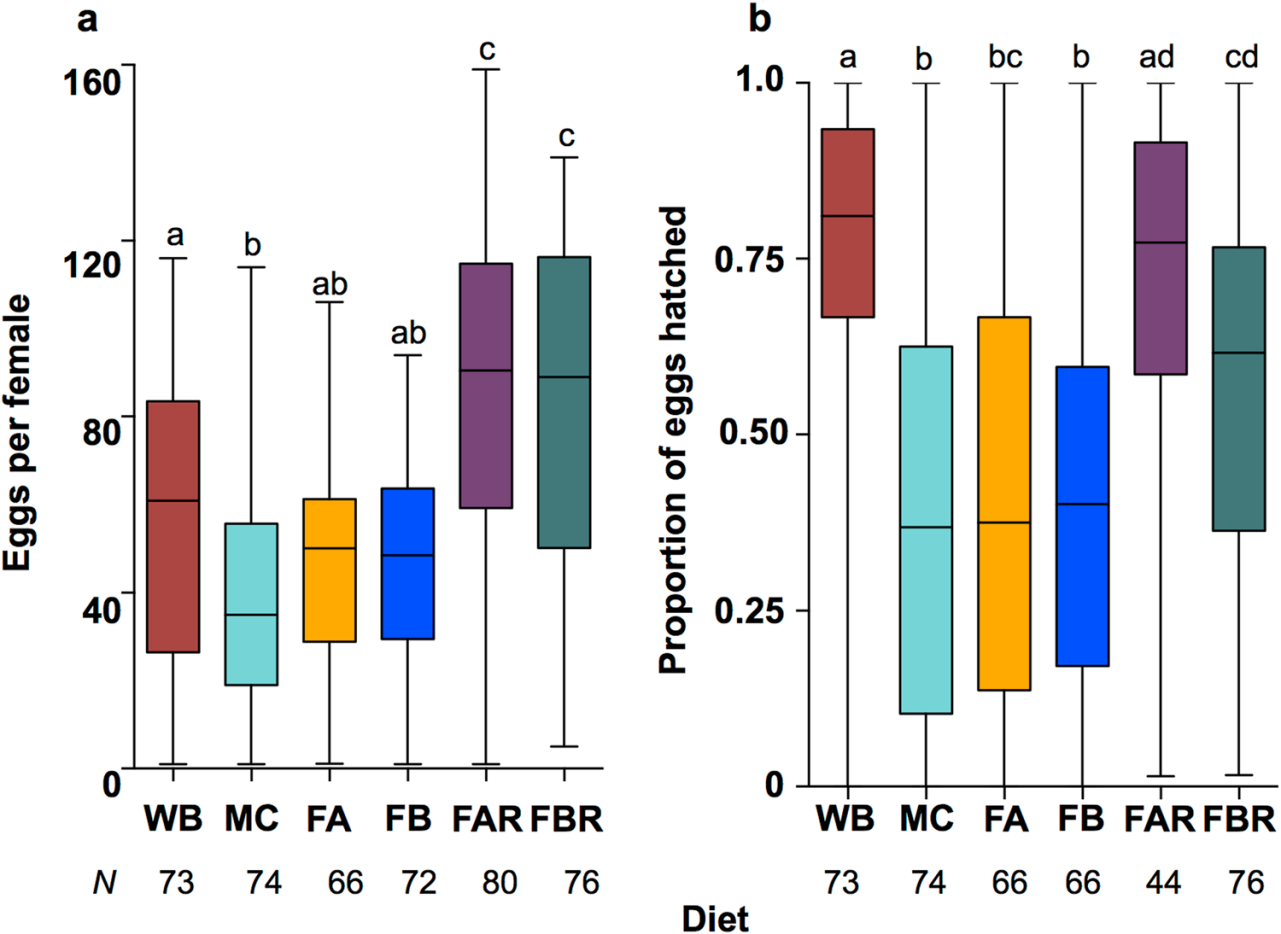
Fecundity and hatch rate for *w*Mel-infected *A. aegypti* fed on diets incorporating infant formula and RBC. Mel mosquitoes were fed whole human blood (WB), or MC protein 125 mg/mL (MC) as control diets. These were compared against two diets that included Alfaré infant formula; FA (15 mg/mL) and FB diets (25 mg/mL), and two further diets that included formula at the same concentrations and 1 mL of Human RBC (FAR and FBR). The FA and FB diets had slightly increased fecundity (**a**), however hatch rates (**b**) were not improved over the MC diet. The FAR and FBR diets had significantly greater levels of fecundity than WB, and the FAR diet had a similar hatch rate. Data were compared using Kruskal-Wallis ANOVA, and Dunn’s multiple comparisons test. Different letter codes represent statistically significant differences between treatments. Box - median and interquartile range. Whiskers - minimum and maximum.

We identified powdered infant formula as a relatively inexpensive, soluble and easy-to-prepare source of carbohydrates, lipids and micronutrients. When this product was fed to mosquitoes at either 15 (Diet F1) or 25 mg/mL (Diet F2) together with MC, we observed greater fecundity than for feeding MC alone (Fig. 3). Although the effect was not statistically significant, this equated to an increase of approximately 5–7 live larvae per female, based on treatment medians. Experiments involving additional concentrations of Alfaré Infant Formula are described in Supplementary File 1 and Supplementary Fig. 3.

When the FA and FB diets were suspended in 1 part human RBC: 2 parts APS (Diets FAR and FBR), we observed significant increases in both fecundity (Fig. 3a, Kruskal-Wallis: *H* = 88.11, *N* = 441, *P* < 0.0001) and hatch rate (Fig. 3b, Kruskal-Wallis: *H* = 98.27, *N* = 399, *P* < 0.0001) compared to MC, and significantly higher fecundity than for WB (Dunn’s test: WB v FAR: *P* < 0.01, WB v FBR: *P* < 0.05). As the hatch rate of the FAR diet was not significantly different to that of WB (Dunn’s test: *P* > 0.05), we selected that diet for further testing.

**Figure 4:**
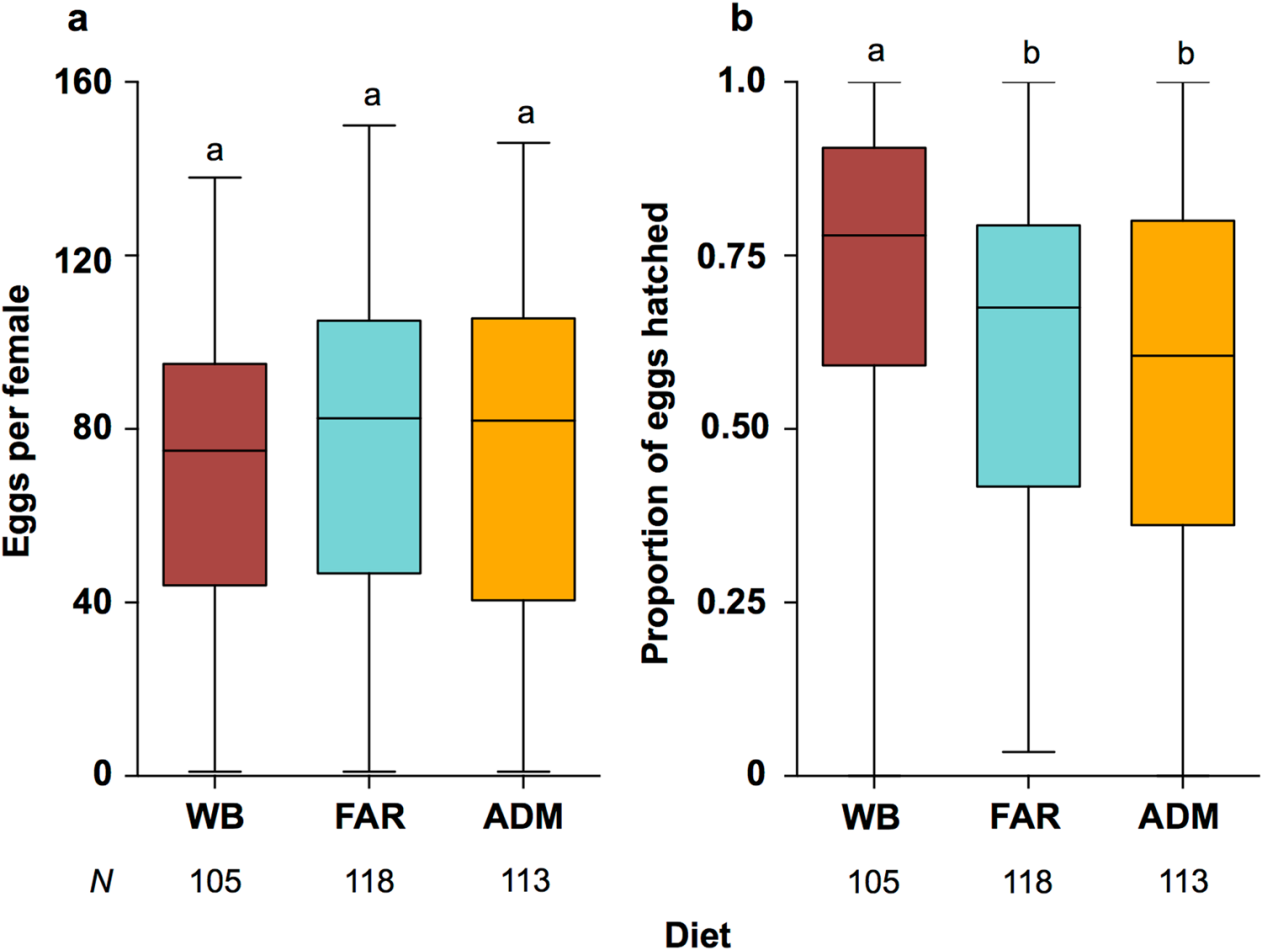
Comparison of Human and Bovine RBC. Mel mosquitoes were fed whole human blood (WB), the FAR diet, or the ADM diet - where human RBC were replaced with bovine RBC. Fecundity (**a**) and hatch rate (**b**) were recorded for individual mosquitoes across 2 experiments. No significant difference in performance was observed between the F1R and ADM diets. Data were compared using Kruskal-Wallis ANOVA, and Dunn’s multiple comparisons test. Different letter codes represent statistically significant differences between treatments. Box - median and interquartile range. Whiskers - minimum and maximum.

One of the issues with the mass rearing of mosquitoes in disease endemic areas is the potential contamination of human bloodstocks. To that end, we tested whether the human RBC in the FAR diet could be replaced with animal RBC, in this case from bovine blood (Diet ADM). In these experiments we observed that both the FAR and ADM diets produced similar fecundity levels to WB (Fig. 4a, Kruskal-Wallis: *H* = 3.21 *N* = 336, *P* = 0.2011). Hatch rates for FAR and ADM were slightly lower than those for WB in these experiments (Fig. 4b, Kruskal-Wallis: *H* = 18.32, *N* = 331, *P* < 0.0001), although they were comparable between FAR and ADM (Dunn’s test: *P* > 0.05), which suggested that bovine RBCs were a suitable alternative. Over these experiments the feeding rate of ADM was 88.65%, which was comparable to feeding rates observed on whole human blood. ADM has an estimated cost of USD $0.03 per mL (See Supplementary File 1), which compares favourably with the cost of previously described BMS^15^. ADM can also be prepared in 10–20 minutes, and the preparation process requires only a scale balance, a centrifuge, and a freezer to store ATP stocks. Details on the composition of all diets are presented in Table 2.

**Table 2:** Composition of diets.

Diet	Components (for 1 mL of diet)	
	*Solids*	*Solutions*
ADM	15 mg Formula, 125 mg MC	0.67 mL APS, 0.33 mL Cow RBC
FA	15 mg Formula, 125 mg MC	1 mL APS
FAR	15 mg Formula, 125 mg MC	0.67 mL APS, 0.33 mL Human RBC
FB	25 mg Formula, 125 mg MC	1 mL APS
FBR	25 mg Formula, 125 mg MC	0.67 mL APS, 0.33 mL Human RBC
MC	125 mg MC	1 mL APS
MCP	125 mg MC	1 mL Human Plasma
MCR	125 mg MC	0.75 mL Human RBC, 0.25 mL APS
MCW	125 mg MC	1 mL Whole Human Blood
PLS	NA	1 mL Human PLS
RBC	NA	1 mL Human RBC
WB	NA	1 mL Whole Human Blood

APS - Aedes Physiological Saline 1X, Formula - Alfaré Infant formula, NA - not present in diet, MC - Concentrated milk protein, PLS - Plasma, RBC - Red blood cells. All diets contained ATP at a final concentration of 2 mM.

### Maternal transmission and *Wolbachia* density

We then started a series of comparative physiological experiments using the F_1_ generation of *w*Mel-infected *A. aegypti* fed either the ADM diet or WB. The purpose of these experiments was to mimic a field release scenario, where laboratory-reared mosquitoes were fed on ADM to generate mosquito material that would be released into the field for mosquito control purposes. The comparison with WB-fed mosquitoes allowed us to examine the effects of ADM on key parameters associated with the fitness of *Wolbachia*-infected mosquitoes. In these experiments we used the MelR line, which was being used for field releases in Brazil (www.eliminatedengue.com/br).

**Figure 5:**
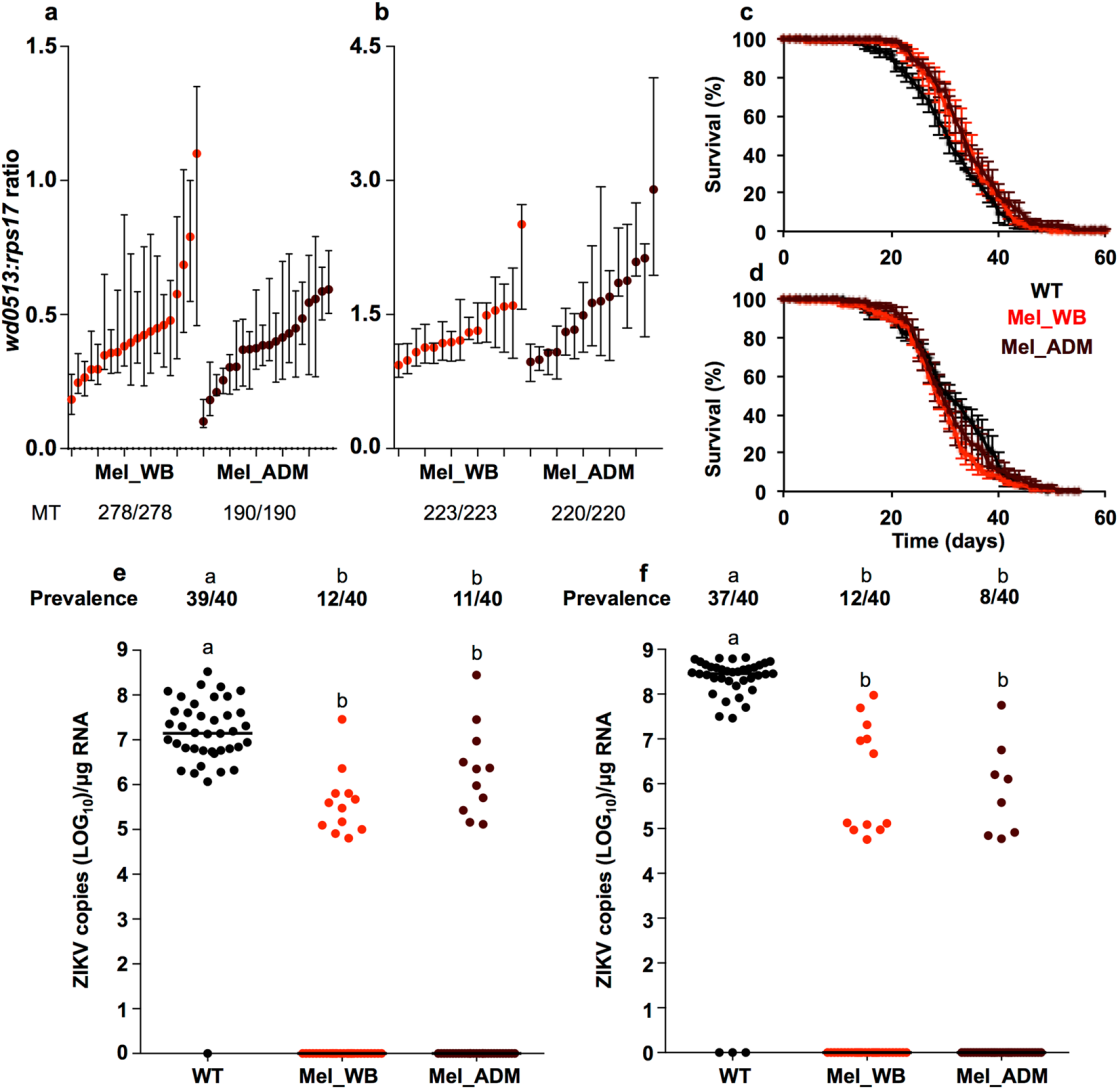
Characterization of the effects of ADM on key traits in *w*Mel-infected *A. aegypti*. Experiments were conducted on either F_1_ (P generation fed either whole blood - Mel_WB (red), or ADM diet - Mel_ADM (crimson)) or F_2_ mosquitoes (where the P generation was fed as above, and the F_1_ generation fed on WB). Some experiments also involved a comparison against WT (*Wolbachia*-uninfected, black) mosquitoes fed on WB. Median (dot) and interquartile range (whiskers) *w*Mel density values (relative to *rps17*) in F_1_ (**a**) and F_2_ (**b**) Mel_WB and Mel_ADM mosquitoes. Each line represents a family of 3–15 adult female mosquitoes. Slightly higher density levels were associated with F_1_ Mel_WB families, and F_2_ Mel_ADM families. MT = Proportion of samples that tested positive for *w*Mel. Data were compared by 2-way ANOVA. (**c & d**) Two independent longevity experiments conducted on F_1_ WT (*Wolbachia*-uninfected), Mel_WB and Mel_ADM mosquitoes. There was no decrease in longevity associated with feeding ADM. Data were compared using Mantel-Cox test. Prevalence (proportion infected) and viral load (number of viral copies) at 7 (**e**) and 14 (**f**) days after oral infection with ZIKV for F_1_ WT, Mel_WB and Mel_ADM mosquitoes. The latter two treatments showed similar levels of resistance to ZIKV infection. Data were compared using Fisher’s exact test, and Mann-Whitney U test. Different letter codes represent statistically significant differences between treatments.

We compared the maternal transmission rate and *Wolbachia* density for F_1_ Mel_WB and Mel_ADM adult females, in a family design. 100% of the mosquitoes in the experiment tested positive for *Wolbachia* through qPCR (Mel_WB - 278/278, Mel_ADM - 190/190). We then compared the bacterial density across families for the two treatments (Fig. 5a) and observed that diet had a significant effect on *Wolbachia* density (2-way ANOVA: *F* = 13.17, *P* = 0.0003), but that this effect accounted for only 2.04% of the variation in the data set. Between-family variation was also a significant factor (2-way ANOVA: *F* = 6.17, *P* < 0.0001), accounting for 18.14% of the total variation (See Supplementary File 1). Comparison of the WB and ADM data revealed that more families with higher median *Wolbachia* density were associated with feeding on WB.

To determine if this effect persisted into subsequent generations, we repeated the assay with F_2_ Mel_WB and Mel_ADM mosquitoes, after the F_1_ adults were fed on human blood. Again, this was modelled on a potential release scenario where released F_1_ generation Mel_ADM mosquitoes would feed on humans encountered in the field. Fecundity levels for the F_1_ generation were not significantly different between the Mel_WB and Mel_ADM lines (See Supplementary Fig. 4, Mann-Whitney U test: *U* = 664, *P* = 0.8277). Maternal transmission was measured for a total of 223 Mel_WB females and 220 Mel_ADM females, across 15 families per treatment. All of these mosquitoes tested positive for the presence of *w*Mel, again indicating 100% maternal transmission. Bacterial density was compared for these families (Fig. 5b), and we observed that both diet and between-family variation were significant factors, with the latter accounting for 33.00% of the total variation. However in this experiment, higher *Wolbachia* density was observed for Mel_ADM families than for Mel_WB families.

### Cytoplasmic Incompatibility

Fecundity and fertility data were compared for 9 reciprocal crosses involving F_1_ WT, Mel_WB and Mel_ADM adult mosquitoes (Table 3). In both predicted incompatible crosses (WT female vs Mel_WB male, and WT female vs Mel_ADM male) we observed an average in excess of 62 eggs laid per female, however all of these eggs failed to hatch. There was a statistically significant difference in hatch rate across the 9 crosses (Kruskal-Wallis; *H* = 148.2, *N* = 311, *P* < 0.0001), however this difference was solely due to the two incompatible crosses, with no difference in hatch rate observed between the other 7 crosses according to Dunn’s multiple comparisons tests (See Supplementary File 1).

**Table 3:** Data from Cytoplasmic Incompatibility experiments.

Female	Male	N (fem)	N (eggs)	Av fecundity	Av hatch rate (%)	Statistical group
WT	WT	41	3422	83.5 ± 4.4	90.2 ± 2.2	a
	**Mel_WB**	**31**	**1951**	**62.9 ± 6.4**	**0.0 ± 0.0**	**b**
	**Mel_ADM**	**27**	**1821**	**67.4 ± 5.1**	**0.0 ± 0.0**	**b**
Mel_WB	WT	36	2510	69.7 ± 3.8	88.0 ± 2.8	a
	Mel_WB	37	2570	69.5 ± 5.4	84.0 ± 3.2	a
	Mel_ADM	41	3009	73.4 ± 4.9	85.0 ± 2.9	a
Mel_ADM	WT	28	2132	76.1 ± 5.3	84.5 ± 4.4	a
	Mel_WB	34	2639	77.6 ± 5.3	87.9 ± 1.6	a
	Mel_ADM	36	2841	78.9 ± 3.8	82.3 ± 2.9	a

Incompatible crosses highlighted in bold. Av = average. N = number of females/eggs. WT = wildtype, uninfected. Mel_WB = *w*Mel-infected mosquitoes, parental generation fed on human blood. Mel_ADM = *w*Mel-infected mosquitoes, parental generation fed on ADM artificial diet. Statistical groups determined by Kruskal-Wallis ANOVA of hatch rate data.

### Longevity

We performed two longevity experiments involving WT, Mel_WB and Mel_ADM mosquitoes, and mortality was monitored daily. In the first replicate (Fig. 5c), WT mosquitoes (*N* = 135) lived an average (±s.e.m.) of 30.69 ± 0.71 days, Mel_WB mosquitoes (*N* = 141) an average of 33.38 ± 0.61 days, and Mel_ADM mosquitoes (*N* = 129) an average of 34.39 ± 0.66 days. Statistical comparison of these groups revealed a significant difference in survival times only between the WT and Mel_ADM groups (Mantel-Cox; *X* ^2^ − 8.788, *P* = 0.003). In the second replicate (Fig. 5d), WT mosquitoes (*N* = 109) lived an average (±s.e.m.) of 31.20 ± 0.80 days, Mel_WB mosquitoes (*N* = 138) an average of 29.23 ± 0.64 days, and Mel_ADM mosquitoes (*N* = 139) an average of 30.93 ± 0.65 days. Statistical comparison of these groups revealed a significant difference in survival times only between the WT and Mel_WB groups (Mantel-Cox; *X* ^2^ − 4.097, *P* = 0.043).

### ZIKV infection

We orally infected WT, Mel_WB and Mel_ADM mosquitoes with ZIKV, and then compared the prevalence of infection (proportion of individuals infected), and viral load (number of viral copies in infected individuals) at 7 (Fig. 5e) and 14 days post-infection (dpi) (Fig. 5f). We observed no significant difference in either prevalence of infection (Fisher’s exact test; 7dpi - *P* = 1.000; 14dpi - *P* = 0.4391) or viral load (Mann Whitney U test; 7dpi - *U* = 35, *P* = 0.595; 14dpi - U = 39, *P* = 0.5208) between the two *w*Mel-infected lines. A comparison of the WT and Mel_WB lines revealed a decrease in prevalence of infection (Fisher’s exact test; 7dpi - *P* < 0.0001; 14dpi - *P* < 0.0001) and viral load (Mann Whitney U test; 7dpi - *U* = 30, *P* < 0.0001; 14dpi - U = 7, *P* < 0.0001) associated with *w*Mel infection. Comparable decreases in prevalence (Fisher’s exact test; 7dpi - *P* < 0.0001; 14dpi - *P* < 0.0001) and viral load (Mann Whitney U test; 7dpi - *U* = 95, *P* = 0.0042; 14dpi - U = 3, *P* < 0.0001) were observed between the WT and Mel_ADM lines.

## Discussion

We have developed a BMS, ADM, which has been designed specifically for use for the mass rearing of *Wolbachia*-infected *A. aegypti* prior to field releases. ADM consists of protein and infant formula, suspended in a mixture of APS and bovine RBC, with ATP used as a phagostimulant. These components are cheap, with the diet costing an estimated USD $0.03/mL of diet. The components are also widely available for commercial purchase from supermarkets and pharmacies in Brazil, with similar products likely to be available in other countries. ADM has an average preparation time of 10–20 minutes, and the preparation process is not particularly arduous, involving no long, complicated experimental procedures, or expensive equipment. Most critically, feeding with ADM leads to the production of large numbers of viable eggs, which suggests that it is a suitable tool for the mass rearing of mosquitoes. Our results suggest that ADM performs comparably with whole human blood, and that it should function as an adequate substitute in situations where large-scale blood feeding is either impossible or impractical.

One of the key aims in the development of this diet was that it be completely free of blood in order to eliminate the need to screen the BMS for human pathogens such as DENV and ZIKV, which could foreseeably be present in blood obtained from disease endemic areas, such as many of the release sites of the EDP. During development of ADM, we saw that feeding a blood-free diet, or an RBC-free diet was severely detrimental to egg production and hatching in *Wolbachia*-infected mosquitoes. While ADM is not completely free of blood, it is free of human blood, which should substantially decrease the risk of viral contamination. Our data showed similar levels of fecundity and egg hatch rate were achieved when either human or bovine RBC were used in the BMS. We hypothesize that the source of RBC could be changed without a great effect on the diet, in the case that a specific type of animal blood could not be sourced, or could not be used for religious or ethical reasons in different regions. However, it should be noted that the use of rodent or avian blood has been shown to have a significant negative impact on the fecundity and fertility of *Wolbachia*-infected mosquitoes^23^, and we do not recommend this for incorporation into a similar BMS.

In our data, we saw that both the PLS and RBC fractions of the blood meal were important to viable egg production in *w*Mel-infected *A. aegypti*. This stands in contrast to the results of similar experiments with uninfected where no eggs were laid after feeding on RBC, and PLS was not associated with a significant decline in hatch rate^26^. While neither assay made a direct comparison of these traits between *Wolbachia*-infected and -uninfected *A. aegypti*, the difference in results could suggest that egg production in *Wolbachia*-infected *A. aegypti* differs from in uninfected mosquitoes. *Wolbachia*-iron interactions appear to be a key component of the *Wolbachia*-host relationship, with *Wolbachia* exhibiting a requirement for iron, altering oxidative stress response in mosquitoes, and increasing host tolerance to iron toxicity^19,27–29^. Our results here could be a further example of this metabolic relationship.

A BMS used with *Wolbachia*-infected mosquitoes must not affect the ability of released mosquitoes to spread into wild mosquito populations, with this ability dependent on high levels of maternal transmission and CI, and high competitiveness with uninfected mosquitoes in the release area. We observed complete (100%) maternal transmission in the F_1_ and F_2_ progeny of *w*Mel-infected females fed on ADM, with in excess of 400 mosquitoes examined. We also observed complete CI when ADM males mated with WT females, and no cost to longevity, fecundity or hatch rate associated with ADM feeding. Together, these data offer no indications that the release of ADM-derived mosquito material would negatively affect the ability of the *Wolbachia* to invade wild mosquito populations.

Another potential consequence of feeding with a BMS was a decrease in bacterial density, which could detrimentally affect the stability of *Wolbachia* infection in a mosquito population, or even lead to less effective interference against key pathogens. To that end, we examined the density of *w*Mel in F_1_ and F_2_ Mel_WB and Mel_ADM adults. Our data highlight the high degree of variability inherent within this trait, with between-family variation in density a significant factor in both experiments. Our statistical models could not explain large proportions of the variation in density, and we hypothesize that this component was likely within-family variation. In both experiments, diet (WB vs ADM feeding) was a significant predictor. However, not only did this variable account for only a small proportion of the total variance in density (F_1_–2.04%, F_2_–3.75%), the effect was not consistent between the F_1_ and F_2_ generations. What these data suggest is that there is unlikely to be a consistent, detrimental effect of ADM on *w*Mel density. However, this would be an important effect to test further in the event this BMS were adopted for mass rearing purposes. Similarly, we saw no evidence that feeding on ADM was associated with a change in the ability of *w*Mel to interfere with ZIKV infection, a critical finding given that the *Wolbachia* transmission blocking strategy is reliant on this phenotype.

It is currently unclear whether ADM is suitable for use with other *Wolbachia*-mosquito host combinations. There could feasibly be difficulties in the application of the diet to other mosquito populations, particularly in those cases where the infecting *Wolbachia* strain has a greater fitness cost. It is also possible that the diet could have utility for mosquitoes that are not infected by *Wolbachia*. However, we recommend that a similar process of characterization be performed before the diet is used with other mosquito populations, including other populations of *w*Mel-infected *A. aegypti*.

The ADM diet appears to have two key shortcomings. The first is the need to include ATP as a phagostimulant, with this component making up approximately 80% of the overall cost. The second is the use of RBC, which will likely limit the storage time of the diet, and could be difficult to source in some locations. However, our results do suggest that RBC or a RBC-derived component is a necessity for a BMS aimed at *Wolbachia*-infected mosquitoes. The identification of a substitute for either of these compounds could greatly improve the diet. Comparative compositional analysis of eggs produced from blood- or ADM-fed *w*Mel-infected *A. aegypti* could further understanding of the effects of *Wolbachia* infection on mosquito egg production. A further avenue of future investigation is to determine whether multigenerational use of ADM is appropriate for *Wolbachia*-infected mosquitoes, or the associated process of adaptation would have negative consequences. The next phase of experiments involving ADM should primarily be focused on applying the diet to a mass rearing setting, and the key issue of diet preservation and storage should also be considered in more depth. Additionally, there is also further scope to investigate the physiological effects of the diet on *A. aegypti* including whether it influences mating competitiveness, and whether egg production in ADM-fed mosquitoes is still comparable to those fed on human blood through multiple gonotrophic cycles. However, in its current state, ADM still represents a critical step forward in the mass rearing of *Wolbachia*-infected *A. aegypti* for disease control purposes.

## Methods

### Mosquito lines and rearing

Mosquito lines used in this project were reared in a climate-controlled insectary (temperature 27 °C ± 1 °C, RH - 70 ± 10%, photoperiod - 12 hours light: dark). Mosquito eggs were hatched in 3 L of RO water containing ½ a tetramin tropical tablet ground into powder (Tetramin). The next day larvae were moved to a fresh tray with the density reduced to 200 in 4 L. Pupae were collected in cups containing no more than 70, and these were transferred to bug dorms (dimensions: 30 × 30 × 30 cm), and allowed to eclose. At 4 days post-eclosion, groups of 50–70 females were transferred to smaller cages (height: 16.5 cm, diameter: 17.5 cm), and starved overnight prior to feeding experiments.

Experiments linked to the development of artificial diets were performed only on a line of *w*Mel-infected *Ae. aegypti* (Mel). This line was derived from the original *w*Mel-infected line developed in Australia^3^, and was then backcrossed into a Brazilian generic background, as previously described^2^. Experiments involving this line took place between G_37_ and G_41_ after the backcrossing was completed. Mel colony mosquitoes were fed on human blood drawn from a willing volunteer, after written consent was obtained. Regular outcrossing of colony material was performed as previously described^22^.

Physiological characterization experiments utilized several mosquito lines. *Wolbachia*-uninfected, insecticide-resistant *Ae. aegypti* (WT) were collected from neighborhoods surrounding Rio de Janeiro, RJ, Brazil starting in late 2015. Each generation 50 F_1_ or F_2_ males were introduced into Mel cages to produce a completely outcrossed *Wolbachia*-infected line (MelR), which has been used for release experiments associated with the World Mosquito Program (www.worldmosquitoprogram.org). MelR and WT adults were fed either whole human blood (WB) or diet ADM (see below) in order to produce eggs. The F_1_ progeny of these lines, named WT, Mel_WB and Mel_ADM were used in experiments described below. Eggs involved in these experiments were stored when nearly dry and used in experiments within a month of being laid.

### Feeding protocol and fecundity and fertility experiments

During the diet development process, Mel mosquitoes were fed using glass feeders, pig intestine and a waterbath. During the physiological characterization experiments, large cages of MelR and WT mosquitoes were fed either blood or the ADM diet using a hemotek system (Hemotek). In all cases, mosquitoes were allowed to feed for 1 to 1.5 hours under low light levels. After the feed, mosquitoes were immediately screened on ice, and non-fed females were removed from cages.

Two days after feeding, Mel mosquitoes were moved to individual opaque plastic cups (height: 5 cm, diameter: 7 cm), containing a piece of damp filter paper on the bottom as an oviposition medium. These cups were placed in closed plastic trays, containing a cup of wet cotton wool in order to maintain high humidity levels. Mosquitoes were provided with 10% sucrose solution *ad libitum*. Females were removed from cups after 4 days. Eggs were then counted and dried slowly over 3 days before being hatched in hatching water (containing ½ of a ground tetramin tropical tablet in 3L). Larvae were counted and removed from cups after 4 days, with this process repeated after a further 3 days. Experiments involving each group of components were repeated 2–4 times.

### Diet components and preparation

We performed a series of fecundity and fertility experiments in order to examine the suitability of different components for use in the development of a BMS. Solid components of these different diets included several types of animal- or plant-derived proteins: Milk C (WPC 80% Nutrifont – Lactallys) or Milk I (Probiotica), Albumin, Pea, Rice, or Soy (Growth Supplements), and Alfaré Infant Formula (Nestlé). The nutritional composition of these ingredients is provided in Supplementary File 2. Solid components were weighed on a Shimadzu AUW220D fine balance. Liquid components included Aedes Physiological Saline (APS) 10X, which was used as the primary solvent for all diets (1L - NaCl - 17.53 g, KCl - 0.5964 g, NaHCO_3_ - 0.0168 g, MgCl_2_ - 0.1143 g, CaCl_2_ - 0.3783 g, HEPES - 11.92 g, pH - 7.0), this was autoclaved and diluted to generate a 1X working solution^26^. APS was allowed to warm to room temperature prior to feeding. Human or Cow blood fractions (Plasma and Red Blood Cells) were obtained by centrifuging whole blood at 1500 rpm for 5 mins. ATP (Sigma Aldrich A2383-5 g, Conc_Final_ = 2 mM) was added to all diets as a phagostimulant^30^. Stocks of APS were stored at 4 °C, while stocks of ATP were stored at −30 °C at a concentration of 100 mM. Diets were prepared on the day of feeding, according to the specifications presented in Table 2. More detail on experiments describing the development stages of the ADM diet is provided in Supplementary File 1.

### Maternal transmission and *Wolbachia* density

MelR mosquitoes were fed either blood or the ADM diet, and then moved into individual cups, as described above. After 4 days, the mothers were removed and stored at −80 °C. Egg numbers were calculated, and then papers dried and hatched independently using the method described above. The mothers were screened for the presence of *Wolbachia* using *w*Mel-specific *wd0513* primers and probe, described previously^7^. Adult mosquito samples were washed in 70% ethanol, and then DNA was extracted in a mixture of 8 parts squash buffer (200 mL stock: 0.242 g TRIS Base, 0.075 g EDTA, 0.292 g NaCl, pH 8.3) to 1 part proteinase K (QIAGEN 19133, 15 mg/mL), with a final reaction volume of 56.25 μL. Samples were heated on a thermocycler at 56 °C for 5 mins, followed by 98 °C for 15 mins, and were stored at 12 °C.

Levels of the *w*Mel-specific gene *wd0513* (NC_002978.6) were then quantified relative to the *Ae. aegypti rps17* gene (XM_001648533) using TaqMan-based qPCR and a Lightcycler 96 (Roche). The mastermix contained the following components per reaction: (DNA - 1 μL, FastStart Essential DNA Probes Master Mix (Roche) - 5 μL, *rps17* primers (10 μM) - 0.3 μL, *wd0513* primers (10 μM) - 0.25 μL, *rps17* probe (10 μM) - 0.06 μL, *wd0513* probe (10 μM) - 0.15 μL, H_2_O - 3.34 μL). Each sample was run in duplicate. The run profile was as follows (Pre-incubation: 95 °C for 10 mins, 45 cycles of 2-step amplification: 95 °C for 15 sec, 60 °C for 30 sec). Primers and probes are described in Supplementary File 1.

Mean normalized expression values for each sample were calculated using qGene^31^. The progeny of mothers that were positive for *Wolbachia* were reared as above, and then sexed and collected at 3–6 days post-eclosion. Up to 15 F_1_ females each from 20 WB-fed mothers, and 20 ADM-fed mothers were screened for *Wolbachia*, as above in order to compare the effect of the ADM diet on the maternal transmission of *Wolbachia*, and adult *Wolbachia* density. In a separate assay, we reared F_1_ Mel_WB and Mel_ADM to adulthood, fed a human blood meal, recorded fecundity, and then conducted a similar experiment comparing the *Wolbachia* density of F_2_ Mel_WB and Mel_ADM adults, examining 15 5 day-old adult female mosquitoes from 15 mothers from each treatment.

### Cytoplasmic incompatibility

Cytoplasmic incompatibility levels were compared between Mel_WB and Mel_ADM mosquitoes, using WT mosquitoes as a *Wolbachia*-uninfected control line. Mosquitoes were reared to pupation as described above. Pupae were then sexed under a stereomicroscope, and then divided into large cages to make up all 9 reciprocal crosses. At 5 days post-eclosion the cages were offered a human blood meal using a hemotek. Unfed females and males were removed from the cages immediately post-feeding. 48 hours later, females were then separated into individual cups (*N* = 27–41 per cross), and fecundity and fertility data were obtained for each cross, as described above. This experiment was repeated twice.

### Longevity

WT, Mel_WB and Mel_ADM eggs were hatched and reared to pupation, as above. Female pupae were sexed using a stereomicroscope, and then moved to small cages, while male pupae were discarded. Pupae were allowed 2 days to eclose, and then cups were removed so that the experiment involved a cohort of similar age. Mortality was recorded daily until day 60, with dead mosquitoes removed from cages daily. Mosquitoes were provided 10% sucrose, which was changed 3 times per week. The assay was run twice, and each experiment involved 3 cages per treatment (*N* = 109–141 mosquitoes per treatment).

### ZIKV infection

WT, Mel_WB and Mel_ADM mosquitoes were reared to adulthood, as described above. At 4 days of age, females were separated into small cages and then starved overnight. Mosquitoes were orally infected with ZIKV using the waterbath feeding system described above. Stocks of ZIKV BRPE (ZIKV/*H. sapiens*/Brazil/BRPE243/2015)^7^ (titer: 3.5 × 10^8^ pfu/mL) were maintained at −80 °C, and defrosted immediately prior to feeding. These were mixed 2:1 with freshly drawn human blood. Mosquitoes were allowed 1 hour to feed, and were then screened for the presence of a blood meal on ice, and all mosquitoes that were not fully engorged were discarded. Mosquitoes were provided with 10% sucrose, which was changed daily. Whole mosquitoes were collected at 7 and 14 dpi, and then stored at −80 °C. Total RNA was extracted using the High Pure Viral Nucleic Acid Kit (Roche), and then total ZIKV (AY632535.2) copies were quantified via RT-qPCR using the Lightcycler Multiplex RNA Virus Master kit (Roche) and a Lightcycler 96 (Roche), as previously described^7^. Two independent infections were performed.

### Statistical analysis

Data for diet development experiments were compiled across experimental replicates and examined jointly. All data were examined for normality using the D’Agostino-Pearson omnibus normality test. Data sets that failed the assumption of normality were examined using non-parametric tests. Fecundity and hatch rate data, including those from the CI experiments, were compared by Kruskal-Wallis ANOVA, and by Dunn’s multiple comparison tests. *Wolbachia* density data were organized by family and diet, and then compared by 2-way ANOVA. ZIKV infection prevalence of infection data were compared via Fisher’s exact test, while ZIKV load data were compared by Mann Whitney U test. Longevity data were compared via Mantel-Cox test. All statistical analyses were performed using Prism V 6.0 g (Graphpad), except for the analysis of the longevity data, which was performed using SPSS V17 (IBM). Statistical output from characterization assays is presented in Supplementary File 1.

### Ethics statement

The human blood used in these experiments was drawn from one willing, adult volunteer by trained medical personnel, after obtaining informed, written consent. This process was conducted according to established guidelines, and approved by The Committee for Ethics in Research (CEP)/FIOCRUZ (License - CEP 732.621). Our use of human blood was in accordance with Brazilian laws 196/1996 and 01/1988, which govern human ethics issues in scientific research in compliance with the National Council of Ethics in Research (CONEP). The bovine blood used in these experiments was produced by Dimeza Alimentos Ltd as a by-product of their operations, and was donated to our group for research purposes, according to the terms of an agreement with Centro de Pesquisas René Rachou.

### Data availability

The datasets generated during the current study are available from the corresponding author on reasonable request.

## Electronic supplementary material

Supplementary materials

Supplementary File 2

Supplementary File 3
