# Supplementary material for: Development and physiological effects of an artificial diet for Wolbachia-infected Aedes aegypti
Source: Sci Rep. 2017 Nov 16;7:15687. doi: 10.1038/s41598-017-16045-6 (PMC5691197; doi:10.1038/s41598-017-16045-6)
Supplement: Supplementary file 1 — Supplementary materials [file 41598_2017_16045_MOESM1_ESM.pdf]

Development and physiological effects of an artificial diet for *Wolbachia*-infected *Aedes aegypti*

Supplementary materials

Heverton Leandro Carneiro Dutra
Silvia Lomeu Rodrigues
Simone Brutman Mansur
Sofia Pimenta de Oliveira
Eric Pearce Caragata
Luciano Andrade Moreira

Supplementary File 1: Additional information

1: Description of additional experiments undertaken during the development of the ADM diet

1.1 - Solvents

Methods:

We performed a pilot experiment comparing different types of solvents in order to see if any were more suitable for feeding as part of an artificial diet. In this experiments all diets included the isolated milk whey protein at a concentration of 150 mg/mL, ATP at a final concentration of 1 mM, and 3 mL of 1 of 5 different solvents. A whole human blood treatment (WB) was used as a control.

The 5 solvents were as follows: (1) 1X APS, as described in the main paper. (2) 1X PBS (NaCl - 136.9 mM, KCl - 2.7 mM, Na_2HPO_4 - 8.1 mM, KH_2PO_4 - 1.5 mM, pH - 7.4), (3) 150 mM NaCl solution, (4). 10% sucrose solution, and (5) Multivitamin solution (0.01524g of ground Centrum Multivitamin in 10mL water). The first two of these were as previously described[1].

Mel mosquitoes were reared to adulthood as described in the main paper, and were offered the artificial diet at 5 days-post eclosion, after approximately 20 hours of starvation.

Results and discussion:

Feeding rates (proportion fed) were as follows:

- WB: 29/32 =0.935
- APS: 24/37 = 0.649
- PBS: 21/42 = 0.500
- NaCl: 15/47 = 0.319
- Sucrose: 27/48 = 0.563
- Vitamin: 19/47 = 0.404

Fecundity data were then obtained following the procedures described in the main paper. In looking at the data for this experiment, we decided to focus on APS for further experimentation as it had the highest feeding rate of the 5 solvents, produced fairly high levels of fecundity, and because it did not contain rapidly perishable organic compounds such as powdered multivitamins or sucrose.

1.2 - Blood fractions and protein

Methods:

After performing the blood fraction experiment described in Figure 1, and the MC protein concentration experiments described in Figure 2 of the main paper, we performed a further group of experiments involving the addition of MC protein at a concentration of 125 mg/mL to whole human blood (WB), human plasma (PLS), or human red blood cells (RBC). WB and MC (MC protein 125 mg/mL) were used as control treatments. Protein-supplemented diets were MCW (+WB), MCP (+PLS), and MCR (+RBC). Blood was separated by centrifugation at 1500 rpm for 5 mins, and then mixed with the protein by vortexing. For MCW and MCP diets, 375 mg of MC was mixed with 3 mL of either WB or PLS. For the MCR diet, the mixture of 3 mL of RBC and 375 mg of protein was too viscous to dissolve fully, so we had to add 1 mL of APS. 3 mL of each of these solutions was fed to Mel mosquitoes on a waterbath system. Mosquito rearing, and fecundity and hatch rate experiments were performed as described in the main paper. Two experimental replicates were performed, and data were compared by Kruskal-Wallis ANOVA and Dunn's multiple comparisons test.

Results and discussion:

We observed that all of the MCW, MCP and MCR had similar levels of fecundity to the WB treatment. Additionally these 3 diets had significantly higher levels of fecundity than the MC treatment, but WB did not (Kruskal-Wallis; $P < 0.0001$). The hatch rate for WB was significantly higher than all other treatments, while the hatch rates of the MCW and MCR treatments was significantly higher than those of the MC and MCP treatments. This suggested to us that the addition of protein to the RBC fraction was sufficient to overcome the decreased fecundity associated with feeding RBC alone (Fig. 2). However, the fact that MCP diet, which consisted of PLS and MC protein, was still associated with a low hatch rate (similar to what was seen when feeding only PLS), suggested that some component in the RBC fraction was still necessary for obtaining a high hatch rate.

1.3 - Additional experiments with Alfaré infant formula

Methods:

We performed a range finding assay to determine the optimal quantity of Alfaré formula to include in our diet. Data on part of these experiments (Diets F1 and F2) have been included in the main paper, however we also examined an additional 2 concentrations of formula (50 mg/mL and 75 mg/mL). Feeding experiments were conducted as described in the main paper. Fecundity data were compared by one-way ANOVA, and Tukey's multiple comparisons test. Hatch rate data were compared by Kruskal-Wallis ANOVA and Dunn's multiple comparisons test. These experiments were repeated twice.

Results and discussion:

While the ANOVA for fecundity data showed there was a significant difference between treatments ($F = 2.784$, $P = 0.0267$), no pairwise comparisons were

significantly significant. However, we did observe a trend towards increased fecundity for the diets with 15 mg/mL (Median - 50 eggs) and 25 mg/mL (Median - 48.5 eggs) formula, compared to diet with only MC protein (Median - 35 eggs). Likewise, there was no significant difference in hatch rate between the treatments, but given the increase in fecundity for the 15 and 25 mg/mL diet, there was an associated increase in the number of larvae produced, when compared to the control diet. For that reason, we chose to include those two diets in the assays involving RBC.

2: List of Primers and Probes

RpS17_F: 5’-TCCGTGGTATCTCCATCAAGCT-3’
RpS17_R: 5’-CACTTCCGGCACGTAGTTGTC-3’
RpS17_Probe: 5’-HEX-CAGGAGGAGGAACGTGAGCGCAG-BHQ-3’

WD0513_F: 5’-CAAATTGCTCTTGTCCTGTGG-3’
WD0513_R: 5’-GGGTGTTAAGCAGAGTTACGG-3’
WD0513_Probe: 5’-FAM-TGAAATGGAAAAATTGGCGAGGTGTAGG-BHQ-3’

ZIKV_F: 5’-TTGGTCATGATACTGCTGATTGC-3’
ZIKV_R: 5’-CCTTCCACAAAGTCCCTATTGC-3’
ZIKV_Probe: 5’-FAM-CGGCATACA/ZEN/GCATCAGGTGCATAGGAG-3IABKFQ3'

ZIKV primers and probe were previously described in [2]

3: Statistical output from characterization assays

3.1 - F_1 *Wolbachia* density 2-Way ANOVA:

Two-way ANOVA	*Ordinary*				
Alpha	0.05				
Source of Variation	*% of total variation*	*P value*	*P value summary*	*Significant?*	
Interaction	7.119	0.0008	***	Yes	
Family	18.14	< 0.0001	****	Yes	
Diet	2.035	0.0003	***	Yes	
ANOVA table	*SS*	*DF*	*MS*	*F (DFn, DFd)*	*P value*
Interaction	2.928	19	0.1541	F (19, 428) = 2.424	P = 0.0008
Family	7.461	19	0.3927	F (19, 428) = 6.176	P < 0.0001
Diet	0.8372	1	0.8372	F (1, 428) = 13.17	P = 0.0003
Residual	27.21	428	0.06358		
Number of missing values	132				

3.2 - F_2 *Wolbachia* density 2-Way ANOVA:

Two-way ANOVA	*Ordinary*				
Alpha	0.05				
Source of Variation	*% of total variation*	*P value*	*P value summary*	*Significant?*	
Interaction	4.088	0.0142	*	Yes	
Family	33.00	< 0.0001	****	Yes	
Diet	3.745	< 0.0001	****	Yes	
ANOVA table	*SS*	*DF*	*MS*	*F (DFn, DFd)*	*P value*
Interaction	9.452	14	0.6751	F (14, 413) = 2.040	P = 0.0142
Family	76.30	14	5.450	F (14, 413) = 16.47	P < 0.0001
Diet	8.659	1	8.659	F (1, 413) = 26.17	P < 0.0001
Residual	136.7	413	0.3309		
Number of missing values	7				

3.3 - Longevity experiment 1 Mantel-Cox test:

	WT		Mel_WB		Mel_ADM	
Line	*Chi-Square*	*Sig.*	*Chi-Square*	*Sig.*	*Chi-Square*	*Sig.*
WT	-	-	3.451	0.063	8.788	0.003
Mel_WB	3.451	0.063	-	-	0.582	0.446
Mel_ADM	8.788	0.003	0.582	0.446	-	-

3.4 - Longevity experiment 2 Mantel-Cox test:

	WT		Mel_WB		Mel_ADM	
Line	*Chi-Square*	*Sig.*	*Chi-Square*	*Sig.*	*Chi-Square*	*Sig.*
WT	-	-	4.175	0.041	0.574	0.449
Mel_WB	4.175	0.041	-	-	2.259	0.133
Mel_ADM	0.574	0.449	2.259	0.133	-	-

3.5 - Cytoplasmic incompatibility Kruskal-Wallis ANOVA

Kruskal-Wallis test	
P value	< 0.0001

Exact or approximate P value?	Approximate
P value summary	****
Do the medians vary signif. (P < 0.05)	Yes
Number of groups	9
Kruskal-Wallis statistic	148.2
Data summary	
Number of treatments (columns)	9
Number of values (total)	311

3.6 - Cytoplasmic incompatibility - Dunn's multiple comparisons test

Number of families	1		
Number of comparisons per family	36		
Alpha	0.05		
Dunn's multiple comparisons test	*Mean rank diff.*	*Significant?*	*Summary*
AA vs. AB	180.7	Yes	****
AA vs. AC	180.7	Yes	****
AA vs. BA	16.14	No	ns
AA vs. BB	38.59	No	ns
AA vs. BC	22.21	No	ns
AA vs. CA	26.59	No	ns
AA vs. CB	35.40	No	ns
AA vs. CC	46.09	No	ns
AB vs. AC	0.0	No	ns
AB vs. BA	-164.5	Yes	****
AB vs. BB	-142.1	Yes	****
AB vs. BC	-158.5	Yes	****
AB vs. CA	-154.1	Yes	****
AB vs. CB	-145.3	Yes	****
AB vs. CC	-134.6	Yes	****
AC vs. BA	-164.5	Yes	****
AC vs. BB	-142.1	Yes	****
AC vs. BC	-158.5	Yes	****
AC vs. CA	-154.1	Yes	****
AC vs. CB	-145.3	Yes	****
AC vs. CC	-134.6	Yes	****
BA vs. BB	22.45	No	ns
BA vs. BC	6.066	No	ns
BA vs. CA	10.45	No	ns
BA vs. CB	19.26	No	ns
BA vs. CC	29.94	No	ns
BB vs. BC	-16.38	No	ns
BB vs. CA	-11.99	No	ns
BB vs. CB	-3.185	No	ns
BB vs. CC	7.497	No	ns
BC vs. CA	4.386	No	ns
BC vs. CB	13.20	No	ns
BC vs. CC	23.88	No	ns
CA vs. CB	8.810	No	ns
CA vs. CC	19.49	No	ns
CB vs. CC	10.68	No	ns

Codes:

AA - WT female x WT male
AB - WT female x Mel_WB male
AC - WT female x Mel_ADM male
BA - Mel_WB female x WT male
BB - Mel_WB female x Mel_WB male
BC - Mel_WB female x Mel_ADM male
CA - Mel_ADM female x WT male
CB - Mel_ADM female x Mel_WB male
CC - Mel_ADM female x Mel_ADM male

3.7 - ZIKV Prevalence of infection Fisher's exact tests:

7dpi WT vs Mel_WB

P value	< 0.0001		
P value summary	****		
One- or two-tailed	Two-tailed		
Statistically significant? (alpha<0.05)	Yes		
Data analyzed	U	I	Total
WT	1	39	40
Mel_WB	28	12	40
Total	29	51	80

7dpi WT vs Mel_ADM

P value	< 0.0001		
P value summary	****		
One- or two-tailed	Two-tailed		
Statistically significant? (alpha<0.05)	Yes		
Data analyzed	U	I	Total
WT	1	39	40
Mel_ADM	29	11	40
Total	30	50	80

7dpi Mel_WB vs Mel_ADM

P value	1.0000		
P value summary	ns		
One- or two-tailed	Two-tailed		
Statistically significant? (alpha<0.05)	No		
Data analyzed	U	I	Total
Mel_WB	28	12	40
Mel_ADM	29	11	40
Total	57	23	80

14dpi WT vs Mel_WB

P value	< 0.0001		
P value summary	****		
One- or two-tailed	Two-tailed		
Statistically significant? (alpha<0.05)	Yes		
Data analyzed	U	I	Total
WT	3	37	40
Mel_WB	28	12	40
Total	31	49	80

14dpi WT vs Mel_ADM

P value	< 0.0001		
P value summary	****		
One- or two-tailed	Two-tailed		
Statistically significant? (alpha<0.05)	Yes		
Data analyzed	U	I	Total
WT	3	37	40
Mel_ADM	32	8	40
Total	35	45	80

14dpi Mel_WB vs Mel_ADM

P value	0.4391		
P value summary	ns		
One- or two-tailed	Two-tailed		
Statistically significant? (alpha<0.05)	No		
Data analyzed	U	I	Total
MelWB	28	12	40

Mel_ADM	32	8	40
Total	60	20	80

3.8 - ZIKV intensity of infection Mann Whitney U tests:

7dpi WT vs Mel_WB

Mann Whitney test	
P value	< 0.0001
Exact or approximate P value?	Exact
P value summary	****
Significantly different? (P < 0.05)	Yes
One- or two-tailed P value?	Two-tailed
Sum of ranks in column A,B	1218 , 108
Mann-Whitney U	30
Difference between medians	
Median of column A	1.441e+007, n=39
Median of column B	347050, n=12
Difference: Actual	-1.406e+007
Difference: Hodges-Lehmann	-1.346e+007

7dpi WT vs Mel_ADM

Mann Whitney test	
P value	0.0042
Exact or approximate P value?	Exact
P value summary	**
Significantly different? (P < 0.05)	Yes
One- or two-tailed P value?	Two-tailed
Sum of ranks in column A,C	1114 , 161
Mann-Whitney U	95
Difference between medians	
Median of column A	1.441e+007, n=39
Median of column C	2.239e+006, n=11
Difference: Actual	-1.217e+007
Difference: Hodges-Lehmann	-8.559e+006

7dpi Mel_WB vs Mel_ADM

Mann Whitney test	
P value	0.0595
Exact or approximate P value?	Exact
P value summary	ns
Significantly different? (P < 0.05)	No
One- or two-tailed P value?	Two-tailed
Sum of ranks in column B,C	113 , 163
Mann-Whitney U	35
Difference between medians	
Median of column B	347050, n=12
Median of column C	2.239e+006, n=11
Difference: Actual	1.891e+006
Difference: Hodges-Lehmann	874463

14dpi WT vs Mel_WB

Mann Whitney test	
P value	< 0.0001
Exact or approximate P value?	Exact
P value summary	****
Significantly different? (P < 0.05)	Yes
One- or two-tailed P value?	Two-tailed
Sum of ranks in column A,B	1140 , 85
Mann-Whitney U	7

Difference between medians	
Median of column A	3.023e+008, n=37
Median of column B	2.405e+006, n=12
Difference: Actual	-2.998e+008
Difference: Hodges-Lehmann	-2.854e+008

14dpi WT vs Mel_ADM

Mann Whitney test	
P value	< 0.0001
Exact or approximate P value?	Exact
P value summary	****
Significantly different? (P < 0.05)	Yes
One- or two-tailed P value?	Two-tailed
Sum of ranks in column A,C	996 , 39
Mann-Whitney U	3
Difference between medians	
Median of column A	3.023e+008, n=37
Median of column C	827163, n=8
Difference: Actual	-3.014e+008
Difference: Hodges-Lehmann	-2.893e+008

14dpi Mel_WB vs Mel_ADM

Mann Whitney test	
P value	0.5208
Exact or approximate P value?	Exact
P value summary	ns
Significantly different? (P < 0.05)	No
One- or two-tailed P value?	Two-tailed
Sum of ranks in column B,C	135 , 75
Mann-Whitney U	39
Difference between medians	
Median of column B	2.405e+006, n=12
Median of column C	827163, n=8
Difference: Actual	-1.578e+006
Difference: Hodges-Lehmann	-52550

4: Supplementary Figures & Figure Legends

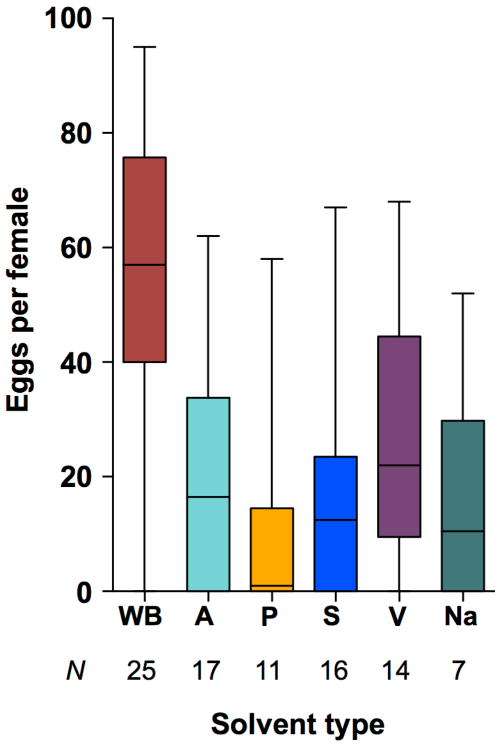

Supplementary Figure 1: Fecundity data for solvents pilot experiment. WB - whole human blood, A - 1X APS, P - 1X PBS, S - 10% sucrose, V - multivitamin solution, Na - NaCl solution. Box - median and interquartile range. Whiskers - minimum and maximum. *N* - number of females analysed in each group.

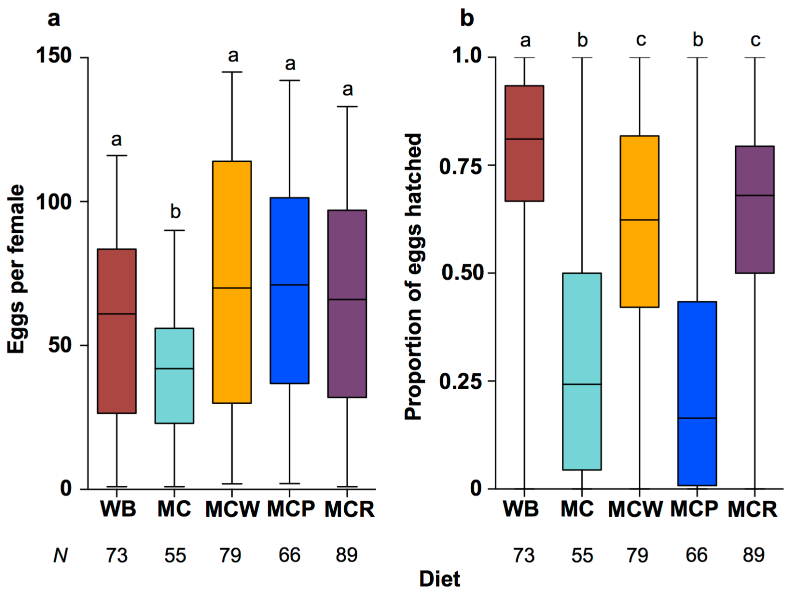

Supplementary Figure 2: Fecundity (a) and hatch rate (b) data for mosquitoes fed on diets containing MC protein and different human blood fractions. Different letter codes represent statistically significant differences between treatments. Box - median and interquartile range. Whiskers - minimum and maximum. *N* - number of females analysed in each group.

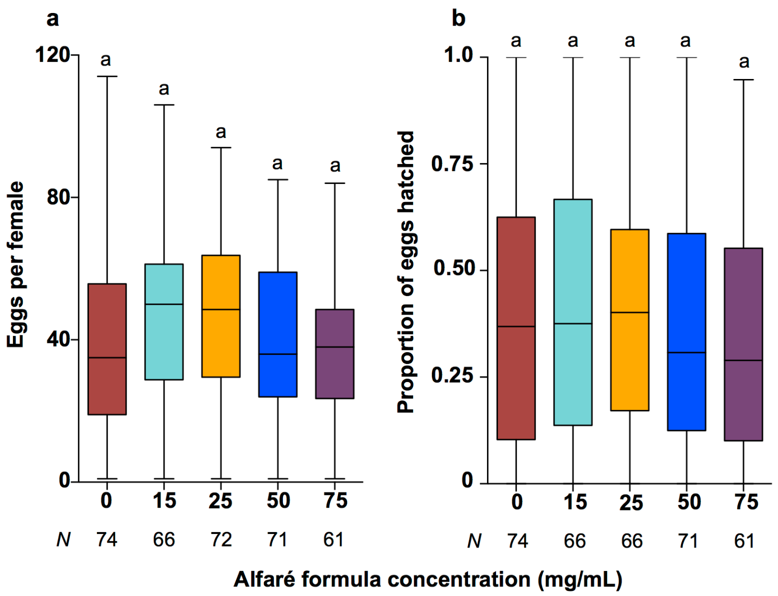

Supplementary Figure 3: Fecundity (a) and hatch rate (b) data for formula concentration range finding assays. Different letter codes represent statistically significant differences between treatments. Box - median and interquartile range. Whiskers - minimum and maximum. *N* - number of females analysed in each group.

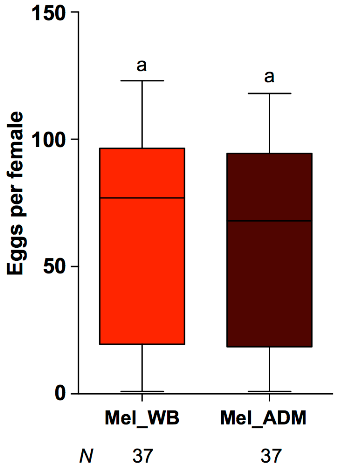

Supplementary Figure 4: Box and whisker plots of fecundity for F_1 Mel_WB (red) and Mel_ADM (crimson) females fed on WB. There was no effect of ADM feeding. Data were compared by Mann-Whitney U test.

5. References:

1 Gonzales, K. K., Tsujimoto, H. & Hansen, I. A. Blood serum and BSA, but neither red blood cells nor hemoglobin can support vitellogenesis and egg production in the dengue vector *Aedes aegypti*. *PeerJ* **3**, e938, doi:10.7717/peerj.938 (2015).

2 Lanciotti, R. S., Kolsoy, O. L., Laven, J. J., Velez, J. O., Lambert, A. J., Johnson, A. J., Stanfield, A. M. & Duffy, M. R. Genetic and serologic properties of Zika virus associated with an epedemic, Yap State, Micronesia, 2007. *Emerg Infect Dis.* **14**, 8, 1232-9, doi:10.3201/eid1408.080287 (2008).
